# Impact of the war in Ukraine on resilience, protective, and vulnerability factors

**DOI:** 10.3389/fpubh.2023.1053940

**Published:** 2023-06-16

**Authors:** Shaul Kimhi, Yohanan Eshel, Hadas Marciano, Bruria Adini

**Affiliations:** ^1^School of Public Health, Tel Aviv University, Tel Aviv, Israel; ^2^Department of Psychology, University of Haifa, Haifa, Israel; ^3^Stress and Resilience Research Center, Tel Hai, Israel; ^4^Stress and Resilience Research Center, Tel-Hai College, Tel Hai, Israel; ^5^Institute of Information Processing and Decision Making, University of Haifa, Haifa, Israel; ^6^Department of Emergency and Disaster Management, School of Public Health, Sackler Faculty of Medicine and ResWell Research Collaboration, Tel Aviv University, Tel Aviv, Israel

**Keywords:** war in Ukraine, resilience, hope, morale, wellbeing, distress symptoms, sense of danger, perceived threats

## Abstract

War or armed conflict is one of the most severe human-made adversities. The current study examines the resilience, protective, and vulnerability factors of a sample of Ukrainian civilians, during the current Russian-Ukrainian war. The level of resilience and coping indicators were compared with the responses of an Israeli sample following an armed conflict in May 2021. The data were collected by an internet panel company. A representative sample of Ukrainian residents (*N* = 1,001) responded to an online questionnaire. A stratified sampling method was employed regarding geographic distribution, gender, and age. The data concerning the Israeli population (*N* = 647) were also collected by an internet panel company during a recent armed conflict with Gaza (May 2021). Three notable results emerged in this study: (a) The Ukrainian sample reported significantly higher levels of the following: Distress symptoms, sense of danger, and perceived threats, compared with the Israeli sample. However, despite these harsh feelings, the Ukrainian respondents reported substantially higher levels of hope and societal resilience compared, to their Israeli counterparts, and somewhat higher individual and community resilience. (b) The protective factors of the respondents in Ukraine (level of hope, wellbeing, and morale), predicted the three types of resilience (individual, community, and social) better than the vulnerability factors (sense of danger, distress symptoms, and level of threats). (c) The best predictors of the three types of resilience were hope and wellbeing. (d) The demographic characteristics of the Ukrainian respondents hardly added to the prediction of the three types of resilience. It appears that a war that threatens the independence and sovereignty of a country may, under certain conditions, enhance the societal resilience and hope of the population under risk, despite a lower sense of wellbeing and higher levels of distress, sense of danger, and perceived threats.

## Introduction

War or armed conflict is one of the most severe human-made adversities. It is frequently accompanied by collateral damage and injuries to the body and mind, destruction of homes and property, significant economic and social costs, and additional long-term negative effects [e.g., (1)]. Studies on the impact of war on psychological resilience indicate the negative effects of distress, anxiety, and uncertainty on individual and social resilience [e.g., (2–4)]. Other studies indicate that in face of stressful conditions (e.g., COVID-19 and armed conflict) resilience is predicted not only by negative effects (such as perceived threats) but also by positive effects (such as hope) (5, 6).

We submit that two major responses (distress and resilience) may simultaneously characterize the Ukrainian population, who face a war that threatens their independence and survivability as a nation (7, 8). Accordingly, as long as hope to survive and successfully overcome adversity still exists in such stressful situations, feelings of resilience and identification with the individual’s country will likely prevail, despite the perceived threat, distress symptoms, and destruction caused to individuals and communities (9). It should thus be expected that the Ukrainians would express a higher level of resilience compared with the Israelis that have been experiencing wars and armed conflicts for over seven decades but do not at present perceive a threat to their independence or survivability as a nation. The investigated Ukrainians are presently affected by the ongoing Russia-Ukraine war which has been prevalent for months and its resolution is not as yet foreseen. Accordingly, to better understand the level of resilience as well as coping factors in Ukraine, we compared results in Ukraine, with results from Israel, measured during a recent armed conflict with Gaza.

Periods of war or armed conflicts elicit among civil societies and individuals varied coping mechanisms, which are aimed at assisting the population and the society to continue their functioning. In the scientific literature, one can find several protective and vulnerability factors of people and countries during, and after a crisis or adversity (3, 10). One conventional way to characterize coping factors is to break them down into protective and vulnerability factors.

*Protective factors* are those indices that the higher they are, the more successful coping they indicate, and vice versa. In the current study, we examine three protective factors: (a) subjective wellbeing, defined as a positive perception of one’s life (11). Earlier studies reported a positive correlation between wellbeing and coping (12). (b) morale is defined as a general term for positive feelings about the prescribed activities of the group (13–15). Earlier studies have found that the level of morale was correlated significantly and negatively with a sense of danger and depression and positively with individual and national resiliencies (16). (c) Hope is the combination of an aim to meet set objectives with the perceived capacity to invest the required energy that is needed to do so (17, 18). Earlier studies reported a positive correlation between hope and good coping (Macriano et al., 2021).

*Vulnerability factors* are those variables the higher they are, the more they decrease coping and the less successful function they indicate, and vice versa. In the current study, we have measured three such factors: (a) Distress symptoms [anxiety and depression; (19, 20)]. (b) A sense of danger regards the extent to which the individual perceives his or her immediate environment as endangering his/her life and/or the life of those closest to him/her (21). (c) Perceived threats are cognitive appraisals of the personal significance of any event threatening his/her wellbeing (22, 23). All three vulnerability factors were found in earlier studies as negatively associated with good coping [e.g., (24)].

Both protective and vulnerability factors have been found to impact the coping and functioning of populations faced with varied stressors. A positive association was found between wellbeing and optimistic sentiments (25, 26). A positive correlation was also found between hope and wellbeing, as well as between hope and resilience (5, 27–29). (30) and Arampatzi et al. (17), have argued that hope and resilience are closely aligned constructs, as they both include a tendency toward maintaining an optimistic outlook in face of adversity. Solomon and Prager (31) have shown that a sense of danger is negatively associated with the perception of control duringadversity.

Many studies have presented that resilience—the ability to recover and return to effective functioning, during and following different adversities, may be associated with the above-mentioned coping mechanisms (2, 32, 33). These associations have been found concerning the capacity of the individual [i.e., –individual resilience; (34–36)], the community [i.e., community resilience; (37)], and the society [i.e., societal resilience; (38)] to recover following adversities. Previous findings have shown that individual resilience is significantly and positively associated with hope and morale, during periods of war or terror events (2, 39).

However, an examination of the relevant literature indicates that only a rather small number of studies have empirically investigated *societal resilience* and associated it with antecedent variables [e.g., (40–42)]. These data suggest that SR levels may reflect environmental conditions and demographic attributes, as well as social and psychological factors. Furthermore, SR reflects political-psychological attitudes such as the strength of democracy and trust in leadership among the population of the investigated society (43). For example, Kimhi et al. (44, 45) demonstrated the validity of the societal resilience scale comparing Israel, the Philippines, and Brazil. Research has indicated that SR is positively associated with wellbeing, higher economic status, trust, and, social norms and negatively associated with psychological distress and exposure to adverse security experiences (40, 45). Additionally, SR was found to be positively associated with both individual and community resilience [e.g., (46)].

Three main objectives of the current study were: (a) to identify the level of individual, community, and societal resilience, as well as protective and vulnerability factors among the Ukrainian population. (b) To compare the results among the Ukrainian population with results of a former sample of Jewish Israelis, measured during a recent armed conflict. (c) To examine to what degree the contribution of each of the protective and vulnerability factors, as well as the demographic characteristics, predict the three types of resilience among the Ukrainian sample.

Based on the findings of previous studies [e.g., (2)] we assume that the three protective factors (subjective wellbeing, morale, and hope) and three vulnerability factors (distress symptoms, sense of danger, and perceived threats) will significantly and positively associate with each other. Additionally, we expect that the three protective factors will significantly and positively correlate with the three types of resilience, while the three vulnerability factors will negatively and significantly correlate with the three types of resilience. Considering the essential differences between the war in Ukraine and the armed conflict between Israel and the Palestinians, we assumed that the Ukrainians would report a higher societal resilience compared with the Israeli sample. The question of the relative importance of the examined variables is presented as an open-ended research question, as we could not find previous studies that have examined this issue.

## Methods

### Sample and sampling

The study was conducted among a sample of the Ukrainian population, living in all Ukrainian regions, except for Crimea and the areas that were at the time under the occupation of the Russian army (Donetsk and Lugansk regions). Data collection was performed by a Ukrainian internet panel company, sampling the varied sectors of the population (*N* = 1,001). The characteristics of the respondents are presented in Table 1. The data collection was conducted on 22–28 July 2022, about 5 months after the invasion of Ukraine, which took place on 24 February 2022. The findings regarding the level of resilience and coping factors were compared to a sample of Israeli civilians (*N* = 647), that was assessed in May 2021, during an active conflict between Israel and Gaza (Operation Guardian of the Walls, 10–21 May 2021), that was characterized by thousands of rocket attacks on civilian communities (47).

**Table 1:** Demographic characteristics of the Ukrainian (*n* = 1,001) and Israeli sample (*n* = 647).

		Ukraine			Israel			
Variable		*N*	%	*M* (*SD*)	*N*	%	*M* (*SD*)	*p*
Age groups	18–25	139	13.9	37.26 (9.56)	18	3	48.52 (26.79)	
	26–35	279	27.9		60	9		12.56***
	36–45	351	35.1		188	29		
	46–55	232	23.2		249	39		
	56 and older	–	–		132	20		
Education	Elementary	3	3	4.03 (0.95)	–		This item was not included	
	High school	52	5.2		–			
	More than high school	264	26.4		–			
	Bachelor’s degree	278	27.8		–			
	Master’s and above	404	40.4		–			
Gender	Male	489	48.9		350	46	2.48 (1.26)	
	Female	512	51.2		297	54		
Family income	Below	494	49.4	2.49	154	53		−0.175
	Average	335	33.5	(1.04)	154	24		
	Above	173	17.3		54	24		
Political attitudes	Left	32	3.2	The majority did not respond	77	11	3.48 (1.70)	
	Center	237	23.7		236	37		
	Right	107	10.7		334	52		
	Difficult to answer	625	62.5		–	–		
Religiosity	Secular	221	22.1	2.05 (0.73)	328	51	1.79 (0.96)	−6.22***
	Traditional	531	53.1		177	27		
	Religious	226	22.6		91	14		
	Orthodox	232	23.2		51	8		
Currently living	In the same place as before	778	77.7		–			
	Moved to another place in Ukraine	223	22.3		–			

### Tools

The study was based on a structured questionnaire that was ethically approved by the Ethics Committee of Tel Aviv University (#0005146–1 from July 12th, 2022). The scales used were validated tools that were shortened for this study. Shortening the scales was done based on the calculation of the reliability of each scale and the effect of excluding items on the Alpha Cronbach, using data that was previously collected with the longer versions. Only scales that proved to be highly reliable after shortening was used.

The questionnaire included the following sections:

**(1) Distress symptoms** (BSI) (48). Four items relating to anxiety (for example: “I feel such restlessness that it is impossible to sit in one place”) and 4 items relating to depressive symptoms (such as “I feel a lack of interest in my world”) were included, ranked on a 5 point Likert scale, ranging from 1 = not at all to 5 = to a very large extent. The internal reliability, measured by Alpha Cronbach of the scale was very good (*α* = 0.89).

**(2) Wellbeing** (49). Five items (for example: “What is your work life at present”) were included, ranked on a 6-point Likert scale, ranging from 1 = very bad to 6 = very good. The internal reliability, measured by Alpha Cronbach of the scale was good (*α* = 0.78).

**(3) Societal resilience** (41). Ten items (such as Ukraine is my home and I do not intend to leave it”) were included, ranging from 1 = strongly disagree to 6 = strongly agree. The internal reliability, measured by Alpha Cronbach of the scale, was excellent (*α* = 0.91).

**(4) Community resilience** (50). Nine items (such as: “I can trust people in my community to come to my aid in case of crisis”) were included, ranging from 1 = do not agree at all to 5 = agree to a very large extent. The internal reliability, measured by Alpha Cronbach, of the scale was excellent (*α* = 0.90).

**(5) Individual resilience** (51). The two items (such as: “I can adapt when changes occur”) suggested by these authors were used, ranging from 0 = do not agree at all to 5 = agree to a very large extent (for the analysis of the data we have re-coded the scale to 1–5). The internal reliability, measured by Alpha Cronbach, of the scale was acceptable for two items scale (*α* = 0.67).

**(6) Hope** (52–54). Three items were adapted in their context to a security threat (for example: “I have hope that I will emerge strengthened from the Ukraine war”), ranging from 1 = very little hope to 5 = very much hope. The internal reliability, measured by Alpha Cronbach, of the scale was very good (*α* = 0.80).

**(7) Morale**. We have used one item: “What is your morale (personal mood) these days?” The answer to the question was given on a 5-point Likert scale, ranging from 1 = very bad to 5 = very good.

**(8) Sense of danger** (16, 30). Four items were included (e.g., “To what extent do you feel that your life is in danger due to the war in Ukraine?,” ranging from 1 = not at all to 5 = to a very large extent. The internal reliability, measured by Alpha Cronbach, of the scale was found to be acceptable (*α* = 0.78).

**(9) Perceived threats** (55). Five items relating to 5 types of adversities were included—economic, social, security, political, and health risks. The responses were given on a 5-point Likert scale, ranging from 1 = not threatening at all to 5 = threatening to a very large extent. The internal reliability, measured by Alpha Cronbach, of the scale was found to be good (*α* = 0.84).

**(10) Demographics.** Characteristics included 13 items: age, gender, socio-economic status, education, political attitudes, level of religiosity, level of exposure to the war, and more.

## Results

We examined the correlations among all the research variables (Table 2). As expected, all the protective factors were significantly and positively correlated with each other, and all the vulnerability factors were significantly and positively correlated with each other. In addition, the three types of resilience significantly and positively correlated with all three protecting factors, and significantly and negatively correlated with two vulnerability factors (distress symptoms and perceived threats). The protective and vulnerability factors (except for a sense of danger) significantly and negatively correlated with each other. A sense of danger was not associated with any of the resilience indices or the level of hope and was only negatively associated with wellbeing and morale, and positively associated with distress symptoms.

**Table 2:** Correlation matrix among the Ukrainian research variables (*N* = 1,001).

Variable	1	2	3	4	5	6	7	8	9
1. IR	–	0.286***	0.200***	0.324***	0.316***	0.305***	−0.284***	−0.067	−0.113***
2. CR		–	0.621***	0.319***	0.215***	0.397***	−0.130***	0.022	−0.108***
3. SR			–	0.304***	0.178***	0.593***	−0.105***	−0.004	−0.167***
4. Wellbeing				–	0.452***	0.393***	−0.390***	−0.177***	−0.265***
5. Morale					–	0.244***	−0.578***	−0.339***	−0.393***
6. Hope						–	−0.185***	−0.012	−0.142***
7. Distress symptoms							–	0.443***	0.479***
8. Sense danger									0.526***
9. Perceived threats									–

***p < 0.001. Shadowed cells represent nonsignificant correlations.

To better understand the signification of the three types of resilience, as well as protective and vulnerability factors in the Ukraine sample, we compared their results with the same variables of an equivalent Israeli sample, measured during Operation Wall Guard (armed conflict between Israel and Gaza strip that took place in May 2021). We have used analysis of variance (ANOVA) to compare all means of the relevant measurements of both samples. Results of these comparisons (Table 3) indicated the following: (a) The Ukrainian respondents reported a significantly higher level of societal resilience (medium size effect), and community, and individual resilience (small size effects), compared with the Israeli respondents. (b) The Ukrainian respondents reported a significantly lower level of wellbeing (large size effect) and morale (medium size effect), compared with the Israeli sample. In contrast, the Ukrainian respondent reported a significantly higher level of hope (medium-size effect), compared with the Israeli sample. (c) The Ukrainian respondents reported a significantly higher level of vulnerability factors, compared with the Israeli sample: distress symptoms and sense of danger (large-size effects), and perceived threats (medium-size effect).

**Table 3:** Analysis of variance (ANOVA): comparison of research variables between the Ukraine sample (*N* = 1,001) and Israeli sample (*N* = 647).

Resilience	Ukraine		Israel		Univariate *F*	η^2^	
	*M*	*SD*	*M*	*SD*			
1. SR	4.35	0.97	3.89	0.89	93.16***	0.05	Medium
2. CR	3.40	0.73	3.29	0.93	7.80**	0.01	Small
3. IR	3.65	0.75	3.56	0.88	4.11*	0.01	Small
4. Wellbeing	3.56	0.92	4.41	0.93	328.97***	0.17	Large
5. Hope	3.95	0.92	3.50	0.96	89.21***	0.05	Medium
6. Morale	2.90	0.78	3.33	0.98	96.58***	0.06	Medium
7. Distress	2.94	0.88	2.23	1.00	230.78***	0.12	Medium
8. Sense of danger	3.70	0.77	2.45	0.91	890.17***	0.35	Large
9. Perceived threats	3.29	0.84	2.79	0.83	136.08***	0.08	Medium

*p < 0.05, **p < 0.01, ***p < 0.001.

η^2^ = 0.01 indicates a small effect. η^2^ = 0.05, indicates a medium effect. η^2^ = 0.14 indicates a large effect.

### Prediction of resilience among the Ukraine sample

**Figure 1:**
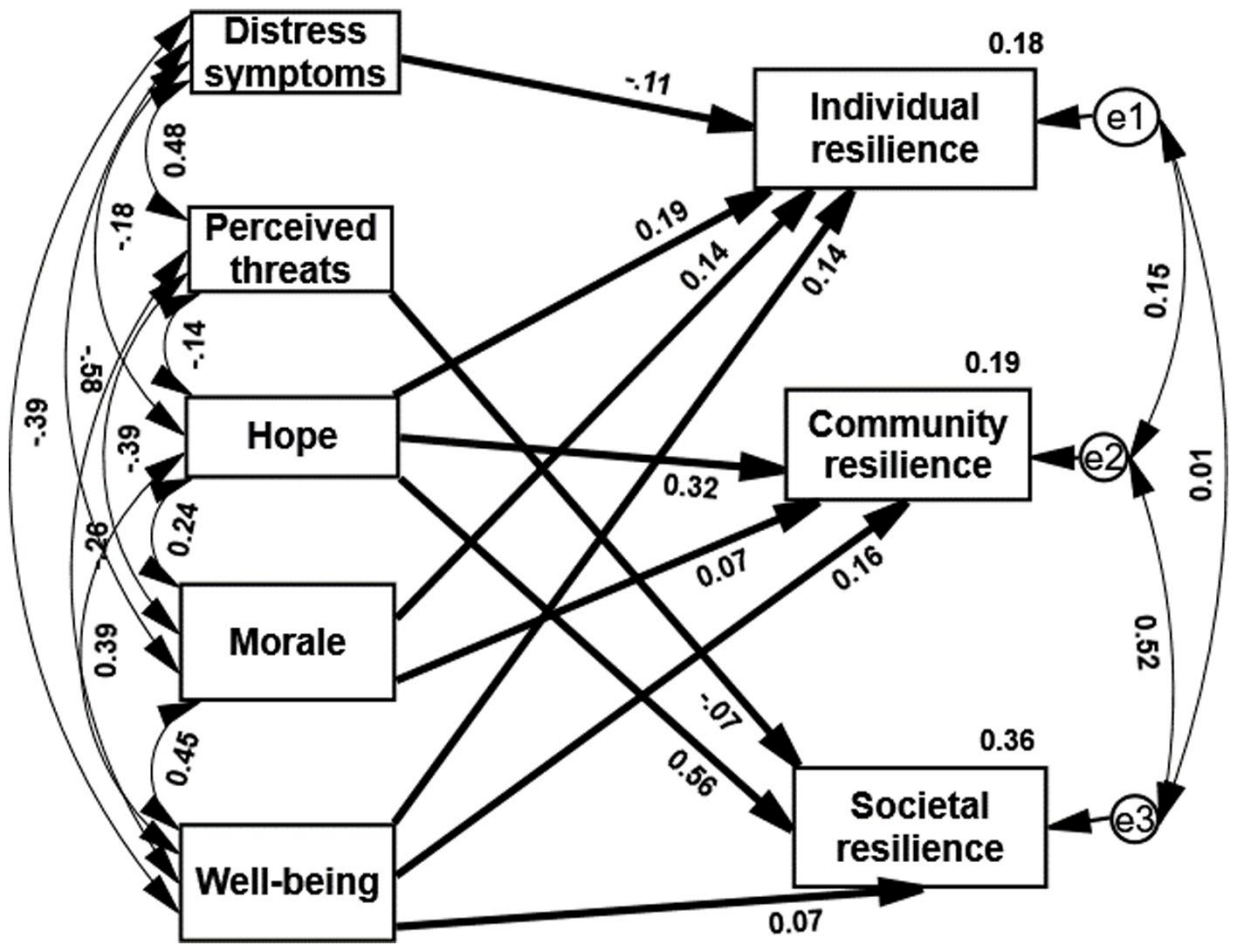
Protective and vulnerability factors, significantly predict three types of resilience among the Ukrainian sample (*N* = 1,001). All the paths in the above figure are significant (*p* < 0.05).

Using Path Analysis (56), we have examined six coping factors (three protecting and three vulnerability), controlling for each other, as predictors of individual, community, and societal resilience. We have analyzed this prediction in two steps: (1) We have used a saturated model (the fit model was good). Results indicated the following: Hope and wellbeing predicted significantly the three types of resilience, morale significantly predicted IR and CR, perceived threat significantly predicted SR and distress symptoms predicted significantly IR. (2) We eliminated all the non-significant paths and ran the path analysis again. Results indicated that a sense of danger did not significantly predict any of the three types of resilience and was eliminated from the analysis. The updated path analysis indicated the following results (Figure 1): (a) Hope is the best predictor of SR, CR, and IR: the higher the hope, the higher resilience reported. (b) Wellbeing significantly predicted SR, CR, and IR: the higher level of wellbeing, the higher resilience reported. (c) Morale significantly predicted IR, and CR: the higher level of morale, the higher resilience reported. (d) Distress symptoms significantly predicted IR: the higher distress, the lower IR reported. (e) Perceived threat significantly predicted SR: the higher the perceived threat, the lower SR reported. (f) The five predictors together explained 18% of IR, 19% of CR, and 36% of SR variability. (g) Overall, according to this analysis, it is possible to assess that the protective factors were better predictors of resilience, compared with the vulnerability factors. (h) Results indicated good Model Fit: CMIN = 0.16, NFI = 0.999, FMIN = 0.014, CFI = 0.996, RMSEA = 0.042.

We conducted additional path analysis using demographic characteristics as predictors of the three types of resilience, controlling for each other. Initially, the following demographic variables were examined: religiosity, gender, age, current settlement size, family status, region of living (currently), average family income, region of living before the war, and the country where the participants have been born. Next, any demographic variable that did not significantly predict at least one type of resilience was removed from the path analysis equation, resulting in two relevant demographic variables (see Table 4). Results indicated the following: (a) Average family income significantly and positively predicted IR, CR, and SR: the higher the level of income, the higher level of resilience reported. (b) Gender significantly predicted IR and SR: females reported a higher level of SR, compared with males and males reported a higher level of IR, compared with females. (c) Each of the two demographic variables explained only 1–3% of the three types of resilience constructs. Overall, this result indicated that demographic characteristics add very little to the prediction of resilience.

**Table 4:** Standardized estimate of path analysis for two demographic variables predicting three types of resilience.

Predictors	Individual resilience	Community resilience	Societal resilience
Family income	0.14***	0.08*	0.08**
Gender	0.07*	0.04	−0.12***
*R* ^2^	0.03	0.01	0.02

*p < 0.05, **p < 0.01, ***p < 0.001.

## Discussion

One of the interesting and somewhat unexpected results of the current study is the finding that all three types of resilience, societal, community, and individual resilience, of the Ukrainian population, were significantly higher than that of the Israeli population, which was also measured during a period of an armed conflict (May 2021). There are several possible explanations for these results, stemming from the different actual, as well as perceived, characteristics of the two conflicts that the two nations (Ukraine and Israel) were involved in (a) The war in Ukraine is perceived as a war that endangers the sovereignty of Ukraine as an independent country (8), and as such may be considered as “a war of independence” (57) or even “a struggle for survival” [e.g., (58)]. In contrast, the round of fighting in Israel did not endanger its existence and was not perceived by the Israelis as an essential part of the fight for independence or the survival of the nation (59). (b) The war in Ukraine strengthens, among others, the national identity of Ukraine (60) and is a source of pride and identification with the country. The round of fighting in Israel is not related to the Israelis’ identity (61). (c) The war in Ukraine resulted in millions of Ukrainian refugees who were forced to leave their country because of the war (62), while Israelis remain in their homeland. (d) The war in Ukraine continued for almost 6 months without seeing an end, while the round of fighting in Israel lasted only several days. It may be assumed that the prolonged adversity and uncertainty concerning the time and status of its completion, impact the Ukrainians’ perception of the struggle (63). (e) This could also be attributed to the fact that the Israeli population has been coping with the geopolitical conflict for several decades now, which may lead to habituation and an increase in resiliency, compared with the acute threat in Ukraine (64). We conjecture that these characteristics of the war contribute to the relatively high national and communal resilience of the Ukrainians. Further research is needed to support this explanation.

The results of the present study clearly show that the various demographic variables had a very small contribution to the prediction of resilience, compared to the psychological variables and especially the protective factors. Still, these results differ from the results of previous studies which presented that demographic variables, such as political positions, age, and gender) contribute to the prediction of resilience, even though it was similarly shown in those studies that the psychological variables contribute to a higher degree [e.g., (65–67)]. A possible explanation for these results is the high level of threat that was perceived by the Ukrainian respondents, according to which the Russian invasion of Ukraine territories endangers the independent existence of Ukraine (8). We suggest that this high perception of threat is shared by the entire sample, beyond demographic differences such as age, gender, economic status, and education. Considering this, psychological variables such as hope and morale are significant predictors of resilience while demographic characteristics contribute very limited to the prediction of resilience in such extreme war conditions. Further research is required to support the explanation proposed by us.

In the professional literature, there are controversial views regarding the relative importance of predicting resilience variables by protective factors (such as hope and morale) versus vulnerability factors (such as stress symptoms) indices. To a large extent, this issue corresponds to the discussion about negative and positive post-traumatic reactions and the association between them (68). Though some studies revealed that both types of coping mechanisms similarly impact resilience, recent studies have found that hope is a much stronger and more stable predictor of resilience and effective functioning compared with negative constructs, such as fear, in varied misfortunes, such as pandemics and armed conflicts (5, 6).

The current study supports these findings, presenting that the protective factors (such as hope and morale) are better predictors of resilience in stressful adversities, compared with vulnerability factors (such as level of anxiety and depressive symptoms, and sense of danger). We conjecture that during situations of continuous stressors, such as wars or prolonged pandemics, the protective factors enhance the capacity of individuals, communities, and society to maintain optimism concerning their future, and as such, they also better predict their resilience.

Although there are certainly diversities between the two conflicts (the Israeli-Palestinian conflict versus the Ukraine-Russia war), comparing the resilience of the two populations during these adversities is not only important but possible because of the following similarities:

1. Both the Israeli and the Ukrainian societies have had to manage protracted conflicts. Though the Israeli-Palestinian conflict has been going on for a longer period of time, Ukraine has also had to deal with an ongoing conflict with Russia, which started even before the 2014 annexation of Crimea. Thus, both populations have endured many years of erupting violence and instability.
2. Both societies are dealing with security impacts on their population, including direct targeting of civilian communities. The data collected in the study took place (in both countries) during a time of daily disruption of the routine of the civilians, due to shelling, displacement of people, and danger of being harmed by rockets aimed at their cities.
3. Both societies have shown a “sense of community” and solidarity that emerged in response to the immediate threat, evidenced by the communities organizing aid for the internally displaced populations.
4. Both societies have developed and/or used innovative mechanisms in an effort to protect their civil population, whether it’s Iron Dome in Israel to intercept rockets or the newly procured protective equipment and systems that were supplied to Ukraine by varied Western countries.
5. Both societies have shown the utilization of similar coping mechanisms to deal with the conflict, such as hope and communal support. Despite the diversities of the conflicts, the study has highlighted both similarities and diversities among the populations, thus the findings contribute toward an understanding of mechanisms that impact on the resilience of populations in extreme situations.

### Limitations

Several limitations of the current study should be noted as follows: (a) The use of an internet sample does not guarantee a representative sample of the Ukraine population. The age of the respondents (up to 55) and the relatively high prevalence of respondents holding academic education indicate that the current sample is probably not representative. It is suggested that the older population of Ukraine does not have access to computers and/or the Internet. (b) Millions of refugees who were fleeing outside of Ukraine were not included in the current sample, presenting another question regarding the representativeness of the current sample. (c) The present study is based on self-report questionnaires, which may be biased. (d) The results of the present study present associations, rather than causality.

## Conclusion

The present study is of great importance as it examines the effects of an ongoing war on a broad sample of the civilian population in Ukraine, a topic that has not had many research opportunities. It appears that a war that threatens the independence and sovereignty of a country may, under certain conditions, enhance the societal resilience and hope of the population under risk, despite a lower sense of wellbeing and higher levels of distress, sense of danger, and perceived threats. The study also reveals the differences in levels of resilience between two types of wars – a sudden eruption of hostilities (the Ukraine war) versus recurrent conflicts (the wars in Israel). It is recommended that further research be implemented to monitor the levels of resilience over time, throughout the crisis, to ascertain whether the high levels of societal resilience persist over time.

## Data availability statement

The datasets presented in this article are not readily available because the raw data cannot be shared due to the restrictions made by the Ethics Committee. Analyzed anonymized data will be shared upon relevant request. Requests to access the datasets should be directed to BA, adini@tauex.tau.ac.il.

## Ethics statement

The studies involving human participants were reviewed and approved by the Tel Aviv University. The patients/participants provided their written informed consent to participate in this study.

## Author contributions

SK and BA designed the study and collected the data. SK analyzed the data and drafted the initial manuscript. HM and YE reviewed and revised the manuscript. All authors reviewed the final manuscript.

## Conflict of interest

The authors declare that the research was conducted in the absence of any commercial or financial relationships that could be construed as a potential conflict of interest.

## Publisher’s note

All claims expressed in this article are solely those of the authors and do not necessarily represent those of their affiliated organizations, or those of the publisher, the editors and the reviewers. Any product that may be evaluated in this article, or claim that may be made by its manufacturer, is not guaranteed or endorsed by the publisher.
